# The first special issue of *Standards in Genomic Sciences* from the Genomic Standards Consortium

**DOI:** 10.4056/sigs.1493697

**Published:** 2010-12-20

**Authors:** Dawn Field, Renzo Kottmann, Peter Sterk

**Affiliations:** 1 Centre for Ecology & Hydrology, Maclean Building, Benson Lane, Crowmarsh Gifford, Wallingford, Oxfordshire OX10 8BB, U.K.; 2 Microbial Genomics Group, Max Planck Institute for Marine Microbiology and Jacobs University Bremen, D-28359 Bremen, Germany; 3 Wellcome Trust Sanger Institute, Wellcome Trust Genome Campus, Hinxton, Cambridge CB10 1SA, U.K.

Face-to-face meetings play a central role in the birth and maturation of communities. Intensive workshops filled with presentations, discussions and working group meetings have always been at the heart of the activities of the Genomic Standards Consortium (GSC). Such work-driven meetings are a key way in which the GSC fulfils its mission. Similarly, meeting reports provide a key mechanism for preserving and disseminating the consensus built at such meetings as they describe the range of speakers and participants present, topics covered and key outcomes and priorities agreed upon by the community.

This issue contains a total of nine meeting reports, all from workshops held between April and December 2010. The first four describe GSC sponsored events. The first overviews the GSC 9 workshop at the J. Craig Venter Institute (JCVI) in April 2010 [1]. This pivotal meeting in the history of the GSC was the first attempt to organize an open registration meeting (with registration fees) and drew over 90 participants, the largest GSC meeting to date. The second report overviews the GSC 10 workshop held at Argonne National Labs in October of 2010 that focused on the M5 project [2]. The next two reports describe “Metagenomics, Metadata and MetaAnalysis” (M3) workshops held at the Intelligent Systems in Molecular Biology [3] and the International Society for Microbial Ecology meetings [4], respectively in 2010. Such satellite meetings allow the GSC to introduce the consensus-products of the GSC to a wider audience and also to collect essential feedback on what the community needs. As of the GSC 8 workshop, all GSC presentations have also been recorded and uploaded to the SCIVEE site. The GSC is planning its next two workshops at the Wellcome Trust Genome Campus in Hinxton, UK in April 2011 and at the Max-Plank Institute for Marine Microbiology in Bremen, Germany in September 2011.

This issue also contains the first five meeting reports in SIGS contributed by external communities. The first describes a workshop held by the wider genomic annotation community as a step towards organizing a “Critical Assessment of Functional Annotation Experiment” (CAFAE) competition [5]. The next two describe the birth and early efforts to co-ordinate a megasequencing project called the Earth Microbiome Project (EMP) [6,7]. The fourth is the inaugural meeting of the BioSharing community [8]. The fifth describes the second workshop of the “Minimum Information about a Biological or Biomedical” (MIBBI) initiative [9]. The GSC is pleased to be an active member of all of these projects.

We sincerely hope this special issue of meeting reports will pave the way for meeting report contributions from a wider range of related communities dedicated to creating and maintaining consensus-driven science.
